# Context Consistency and Seasonal Variation in Boldness of Male Two-Spotted Gobies

**DOI:** 10.1371/journal.pone.0093354

**Published:** 2014-03-26

**Authors:** Carin Magnhagen, Sebastian Wacker, Elisabet Forsgren, Lise Cats Myhre, Elizabeth Espy, Trond Amundsen

**Affiliations:** 1 Department of Wildlife, Fish, and Environmental Studies, Swedish University of Agricultural Sciences, Umeå, Sweden; 2 Department of Biology, Norwegian University of Science and Technology (NTNU), Trondheim, Norway; 3 Norwegian Institute for Nature Research (NINA), Trondheim, Norway; Liverpool John Moores University, United Kingdom

## Abstract

In order to attribute the behaviour of an animal to its personality it is important to study whether certain behavioural traits show up consistently across a variety of contexts. The aim of this study was to investigate whether breeding state males of the two-spotted goby, *Gobiusculus flavescens*, showed consistent degree of boldness when tested in four different behaviour assays. We also wanted to investigate whether boldness varied over the breeding season in accordance with changes in male-male competition for matings. We used two standard assays (the emergence test and the open field test), and two simple assays related to threat response. Repeated runs of each of the tests were highly correlated, and we found significant correlations between all four assays. Thus, we have documented both a within and a between-context consistency in risk-taking behaviour. Furthermore, we found that goby males studied during the middle of the breeding season were bolder than males studied at the end of the season. Since male two-spotted gobies face strongly decreasing male-male competition as the season progresses, the benefit of being bold for the mating success of the males may differ over the time of the breeding season. The difference in behaviour found over the season thus corresponds well with the sexual dynamics of this model species.

## Introduction

Animal personality is defined as individual consistency in behaviour within and across contexts [1]. This phenomenon is currently receiving much attention, and the adaptive explanation for the coexistence of behavioural types is discussed [2]. Five main axes of animal personality have been defined: boldness, exploration, activity, aggressiveness, and sociability [3], [4]. The shyness – boldness gradient is probably the most commonly studied personality dimension, and has been analysed for many species and taxa since Wilson and co-workers [5], [6] introduced this concept to the field of animal behaviour. In these studies, certain standard behaviour assays have been widely adopted across taxa. One such assay is the emergence test, which measures latency to emerge from shelter [7]–[9]. Other commonly used assays are the open field test, investigating the behaviour of an animal when released into a novel non-structured arena [10]–[12], and the novel object test studying the reaction to an unfamiliar object [13]–[15]. Usually one of these assays is used to quantify an individual's degree of boldness. However, to be able to interpret the test results as indicators of different personalities, one assay may not be enough, as pointed out by Beckmann and Biro (2013) [16] and Carter et al. (2013) [17]. With multiple tests of boldness, it is possible to get a more general picture of how an individual balance risks and gains, to define its personality along the shyness – boldness axes. Consistency in individual boldness across contexts (assays) has been documented in several species [8], [13], [18], [19], but not found in others [16], [20], [21].

In animals where mating success depends on the intensity of courtship or other types of conspicuous activities, the sex competing for access to partners has to balance the prospect of matings with the risk of being detected and caught by predators [22]. Individuals with different personalities may make different trade-offs in their risk-taking during a mating event, which may influence their mating success. A few studies have previously shown that females may choose mates according to boldness [23]–[25]. Many animal species breed repeatedly over the course of a single breeding season. Environmental and social conditions, including the abundance of predators, competitors and availability of mates, may change significantly during this period. If there is a seasonal variation in the intensity of sexual selection, this could affect the costs and benefits of boldness and other personality traits. Hence, temporal variation in the intensity of sexual selection may maintain a variation of personalities in the population and/or select for individual plasticity in behaviour [26].

Previous work has analysed changes in risk-taking over the breeding season as an effect of the decrease of future reproductive prospects with time [27]–[29]. Our study species, the two-spotted goby (*Gobiusculus flavescens*), has a life-span of one year only, and spawns repeatedly over one breeding season [30]. It has a dramatic variation in sexual dynamics over the breeding season, with strong male-male competition for matings in the beginning of the breeding season and a near-absence of male competition at the end of the season [31], [32]. This is probably due to higher mortality rates in males compared to females, reversing the operational sex ratio from male- to female-biased (Forsgren et al. 2004). Theories on cost of reproduction predict that investments in reproduction should increase with a decrease in future reproductive opportunities [33]. However, the benefits of being bold during the reproductive season should also depend on the strength of male-male competition, which would in the two-spotted goby counter the predictions of increased investment with time, as competition decreases over the season [31], [32]. The aim of this study was first to investigate whether four different boldness assays give consistent interpretation of individual boldness in breeding state males, compared within and across contexts, thus making it possible to distinguish different personalities among these males. If this was confirmed, we specifically wanted to investigate whether boldness varies over the season in accordance with the sexual dynamics of the species. We predicted a higher boldness earlier in the season when male mating competition is high compared to later when competition is largely non-existent. To our knowledge, this is the first study to investigate changes in boldness in relation to a predictable temporal change in mating competition.

## Materials and Methods

### Ethics statement

The experiments in this study comply with the current laws of Sweden and were approved by the Gothenburg Ethical Committee on Animal Research (licence no. 166–2008). Collection of fish along the Swedish coast does not require permission. The field collections did not involve endangered or protected species. The fish were handled cautiously to minimise stress, in order to retain as natural behaviour as possible.

### Study species

The two-spotted goby is a semi-pelagic shoaling fish commonly occurring in kelp forests along shallow rocky areas along the shores of Northern Europe [34]. During the breeding season (May – July) males defend nests in seaweed or empty mussel shells [35], [36]. Males, with bright nuptial coloration, attract females to spawn by a suite of courtship displays [37], [38]. Females attach the eggs to the nest substrate during spawning, and males care for the brood until hatching [39], [40]. During the breeding season, reproductive males are solitary and have to take risks during courtship in the open water column. The two-spotted goby typically lives in in kelp forests, a highly fragmented habitat and has to leave shelter to feed and court [41]. The species is an important prey item for young piscivorous fishes, including Atlantic cod *Gadus morhua* [42]. In particular, solitary breeding males may face high mortality during their single reproductive season [31], but can breed multiple times if not taken by predators [30]. Thus, variation in boldness is likely to have significant fitness effects, making the species a suitable model for investigating individual boldness.

### Fish collection and maintenance

The study was carried out in June-July 2009, at Sven Lovén Centre for Marine Sciences, Kristineberg (58°15′ N, 11°27′ E), on the west coast of Sweden. The study consisted of two parts, conducted three weeks apart, starting 3 and 24 June, respectively. Stationary male two-spotted gobies (i.e. males likely to have a nest) were caught, using dip nets while snorkelling, just before each of the two runs of observations. Different fish were used during the two time periods, but were collected in the same location, close to the field station. After being captured, the fish were immediately placed in individual containers. The fish were then transported by boat to the laboratory, where they were housed in individual aquaria (30×20×30 cm; hereafter termed ‘home aquarium’), with gravel on the bottom and artificial seaweed as shelter. The aquaria were located in a thermo-controlled room with air and water temperatures of 16°C, and a photo-period of 14L∶10D, and all tests took place in this room. The water temperature in the species' breeding habitat fluctuates depending on weather conditions, with the chosen temperature within the typical temperature range for the study periods. We kept the temperature the same in both study periods, to avoid any confounding effect of temperature on behaviour. In all aquaria used for storage and experiments, the walls were painted brown on the outside, to reduce mirror effects of the glass. In the home aquaria one wall was kept transparent, to allow for observations from the side. The fish were acclimated for 48 h before the start of the first boldness assay. Food (*Artemia* spp. nauplii) was provided ad libitum in the morning and afternoon. Tests were always conducted at least two hours after feeding. The procedure for the collection of fish and the laboratory conditions were the same during the two study periods.

To test for individual consistency of behaviour within and between contexts, two commonly used boldness assays were performed (emergence test and open field test) as well as two tests performed in connection with catching and feeding of the males in their home aquaria. Each individual was tested at least twice with each assay. (see Table 1 for order and timing of assays).

**Table 1:** Order and timing of tests and behaviour data used in subsequent analyses of boldness in two-spotted goby males after 48 h of acclimatisation in their home aquarium.

Day	Test	Run #	Type of data
1	Catch time	1	Time to catch fish in home aquarium
1	Emergence test	1	Boldness score (PC1)
2	Freeze time	1	Latency to move after food delivery
3	Catch time	2	Time to catch fish in home aquarium
3	Emergence test	2	Boldness score (PC1)
7	Catch time	3	Time to catch fish in home aquarium
7	Open field test	1	Boldness score (PC1)
8	Freeze time	2	Latency to move after food delivery
9	Catch time	4	Time to catch fish in home aquarium
9	Open field test	2	Boldness score (PC1)

### Emergence from shelter

**Figure 1:**
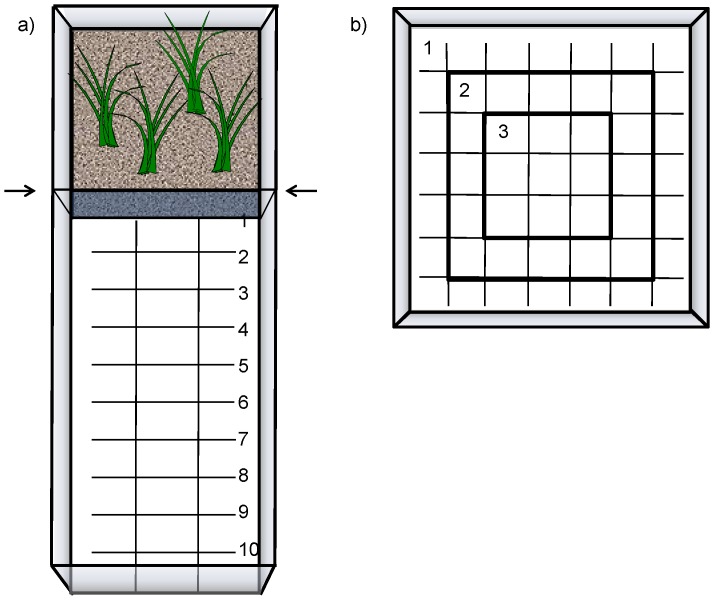
Experimental set-up from above for two boldness assays used on two-spotted goby males. a) Emergence test with sheltered area on top and sliding divider indicated with arrows, distance from divider marked 1–10 with 5 cm between lines, total area 25×75 cm. b) Open field test, showing the areas defined as zones 1–3, grids 6×6 cm, total area 45×45 cm (outermost edges, 1.5 cm, not marked).

The emergence test reveals an animal's propensity to leave a sheltered area to enter into an unknown open space, and is considered to be a measure of boldness [7], [8]. In nature, a two-spotted goby male would have to leave his sheltered nest area among the sea-weed to look for females, and would thereby increase the risk of being detected by predators. The test was conducted in aquaria (75×25×30 cm) that were divided into one smaller compartment with a length of 25 cm and one larger compartment of 50 cm (Figure 1a). The smaller compartment was designed to provide a protected area for the fish. In this compartment, the bottom was covered with gravel, and there were four artificial seaweeds as shelter. Illumination, provided from above, was shaded by a shelf covering the small but not the larger compartment. The larger compartment contained no structural elements and the bottom was transparent with a white surface underneath. A grid (5×5 cm) was painted on the white surface to facilitate recordings of behaviour. This compartment was, due to the lack of shelter, and because it was unknown to the fish at the start of a trial, assumed to be perceived as an unsafe environment by the fish.

A non-transparent divider separated the two compartments when we introduced a fish to the smaller compartment. After 30 min acclimation we carefully lifted the divider about 15 cm, allowing the fish to enter the larger compartment. The larger compartment was filmed from above for 30 min from the moment the divider was lifted. For each fish, we recorded latency to enter the larger compartment, number of times leaving the small for the large compartment (switches), distance moved from the divider (number of grid lines crossed), and time spent in the larger compartment. A bolder fish was expected to enter the large compartment faster, stay longer, and move further away from the sheltered area than a shyer fish.

### Open-field test

The open field test, in which the animal is placed in a novel open environment is commonly used for testing boldness across taxa. Activities typically recorded include distance moved and location in the area (reviewed in [13]). This would resemble a situation in which a fish has been forced into a space in the near-shore open water column, by wave action or by being hunted by a predator [17]. The alternatives of the fish are then to either quickly go to the wall and staying there immobile to avoid detection, or to get familiar with the area by moving around in the central parts of the aquarium. For this assay, the test aquaria (45×45×30 cm) contained no structural elements, and the bottom was transparent with a white surface underneath. A grid (6×6 cm) was painted on the white surface to facilitate recordings of behaviour. The bottom of the aquarium was divided into three zones, to be able to register the fish's location, with zone 1 along the edge, and zone 3 in the centre (Figure 1b). Constant and uniform light was provided from above. Each fish was released by a standardized procedure at the surface, in the centre of the tank (zone 3), carefully submerging the dip-net into the water and allowing the fish to swim out. The behaviour of the fish was then filmed from above for 10 min. The distance moved and the number of visits into the central parts of the tank (zones 2 and 3) from its main position along the edge (zone 1) was expected to reflect the boldness of a fish, since it would be more exposed in the centre as opposed to the edge. We recorded latency to reach zone 1 after release, latency to return to the inner part (zones 2 and 3) and number of times entering zone 2 and 3, respectively. These parameters were recorded for the full 10 min of the recording. Further, general movement was recorded as number of grids crossed during the first two minutes, to assess the immediate behavioural response after the fish had been released into the novel environment.

### Catch time

Correlations between catch order and boldness have previously been shown in other species [43], [44]. Before the emergence and open field tests, the male was carefully caught in its home aquarium with a dip-net and transferred to the experimental aquarium. The same type and size of dip-net was always used, and the observer took care to catch the fish in a standardized manner, not chasing the fish vigorously and not letting its evasive behaviour affect the intensity of catching efforts. Aiming to catch the fish with a dip-net in principle simulates a predation attempt that includes chasing the fish. The time needed to catch the fish was recorded with a stop-watch (to the nearest s). This assay allowed us to analyse boldness-shyness as reflected in evasive behaviour, to test for a potential consistency in evasive behaviour and any relationships between evasive behaviour (catch time) and other assays. The behaviour of the observer was highly unlikely to be influenced by any expectations of behaviour, since behaviour data was not analysed before the experiments were completed.

### Freeze time

Freezing is usually interpreted as an indication of shyness/boldness and occurs in many taxa, likely to decrease attention in the presence of predators [13]. If a sudden disturbance occurs in an aquarium, a two-spotted goby would typically flee and/or “freeze” (stay still) in the artificial algae or on the bottom. This happens, for instance, when something is dropped into the aquarium and creates a small splash at the surface – an event that might mimic a predation attempt by, for instance, a tern. The time elapsing until a fish starts to move again after such an event is likely to reflect boldness. At two feeding occasions during the experiment, food (*Artemia* spp. nauplii in a small volume of water) was dropped in the middle of the aquarium in a standardized manner, from a height of ca 5 cm. When this happened, the fish would always flee and freeze as described above. It is, however, likely that the fish soon detected (by vision, olfaction, or both) that planktonic food was now available in the aquarium. The fish would then leave its shelter and start moving in order to feed. The procedure was video-taped for 10 min, and the time elapsing until the fish started to move was recorded (to the accuracy of 1s). Fish that did not move during the 10 min recording were assigned a notional freeze time of 10 min (two fish did not move during both runs).

### Time of season and study objects

The tests were performed during two separate periods of the breeding season, 5–13 June (mid-season), and 26 June – 4 July (late season). Each of the test periods started with 24 fish, thus, a total of 48 fish were used for the whole study. However, due to mortality (3 fish) and some technical problems with video recordings after the first emergence test, 44 fish were tested twice for this test (20 and 24 for the two periods, respectively), and 38 fish for the open field test (16 and 22, respectively). Thirty-eight fish were successfully tested in both assays (16 mid-season and 22 late season). The fish from the two test periods differed slightly in length (mm ± SD = 44.1±3.5 [mid-season], 46.6±4.0 [late season]; *F*_1,43_ = 4.86 p = 0.033), and weight (mg ± SD: 669±170 [mid-season], 807±195 [late season]; *F*_1,43_ = 6.4, p = 0.015), but not in body condition (estimated as residuals from a length-weight regression; *F*_1,43_ = 1.61, p = 0.21). The fish used represented a typical size spectrum for the species and times of season.

### Data analyses

For the emergence test and the open field test, we performed principal component analyses (PCA) to obtain a single behaviour score for each assay. For the emergence test, the measurements included in the PCA were: (i) latency to enter the larger compartment, (ii) number of switches from the small to the large compartment, (iii) mean distance moved from shelter (number of grid lines crossed) per event, (iv) mean time spent in the larger compartment, and (v) total time spent in the larger compartment. A fish that never emerged was assigned a notional emergence time of 30 min (six fish did not emerge in any of the two runs). For the open field test, measurements included were: (i) time from being released until reaching zone 1, (ii) time until first leaving zone 1, (iii) number of times entering zone 2, (iv) number of times entering zone 3, and (v) grid lines crossed for the first two minutes after release. The principal components explaining most of the variation of the data, and with an Eigenvalue greater than 1, were used as estimates of boldness in further analyses. Time (s) to catch the fish in their home aquarium and latency (s) for the fish to start moving after freezing (see above) were also used as boldness estimates.

The residuals of the data were not normally distributed and we therefore used non-parametric tests. Spearman rank correlation was used to test for within-individual consistency, within and between assays. Wilcoxon matched-pairs test, and Friedman's Anova were used for the individual changes in test response across repeated runs. For the tests mentioned above, comparing within-individual scores, data from the two time periods (mid- and late season) were pooled. Mann-Whitney U-test was used to test differences in boldness between the two time periods. Correlations between assays and differences between time periods were tested using first runs only, thus comparing the response of a novel test, and avoiding the possibility that learning has different effects in the different assays. Further, for the six correlations between assays, the Benjamini-Hochberg procedure for controlling false discoveries [45] was used to correct for multiple testing, as recommended by Waite et al. (2006) [46] for ecological studies. The threshold P-values for rejecting the null-hypothesis of no correlation will then be: 0.0083, 0.0167, 0.025, 0.033, 0.042, 0.05, respectively, ranking the test results from the lowest to the highest P-value [46].

Analyses were performed with Statistica v. 10 (StatSoft, Inc. (2012)).

## Results

### PCA analyses

For the emergence test, the first component (PC1) of the PCA explained 63.5% of the variance. A high PC1 score reflected a quick emergence time, many switches between the shelter and the open habitat, a high number of grid lines crossed, and long time spent in the open area, both in total and per event (Table 2). For the open field test, the first component (PC1) of the PCA explained 54.8% of the variance. A high PC1 score reflected a relatively long time from release until entering zone 1, a relatively quick revisiting of the central parts (zone 2 and 3), entering the central parts many times, and a high swimming activity (i.e. number of gridlines crossed during the first two minutes) (Table 2). Thus, for both assays we interpret the PC1 score as a relevant degree of boldness, while PC2 in both cases were more difficult to interpret, and explained much less of the total variance (ca 19-20%; Table 2).

**Table 2:** Loadings for the two first principal component (PC1, PC2) of the measures included in principal component analyses.

*Assays*/Measurements		PCA loadings	
*Emergence test*	Median (range)	PC1	PC2
Time to emerge (s)	463.5 (0–2100)	−0.82	0.32
Number of switches	6 (0–60)	0.77	−0.54
Lines crossed per emergence	3.5 (0–10)	0.88	0.02
Time out per emergence (s)	11.8 (0–287.5)	0.63	0.75
Total time out (s)	111.5 (0–1725)	0.91	0.20
Variance explained (%)		65.3%	20.1%
Eigenvalue		3.26	1.00
*Open field test*			
Time (s) to zone 1	2 (0–45)	0.53	0.11
Time (s) to leave zone 1	162 (1–600)	−0.64	0.61
Times entering zone 2	2 (0–78)	0.89	0.36
Times entering zone 3	0 (0–32)	0.89	0.41
Lines crossed/2 min	53 (0–117)	0.68	−0.52
Variance explained (%)		54.8%	19.1%
Eigenvalue		2.73	0.95

### Within-context consistency

**Figure 2:**
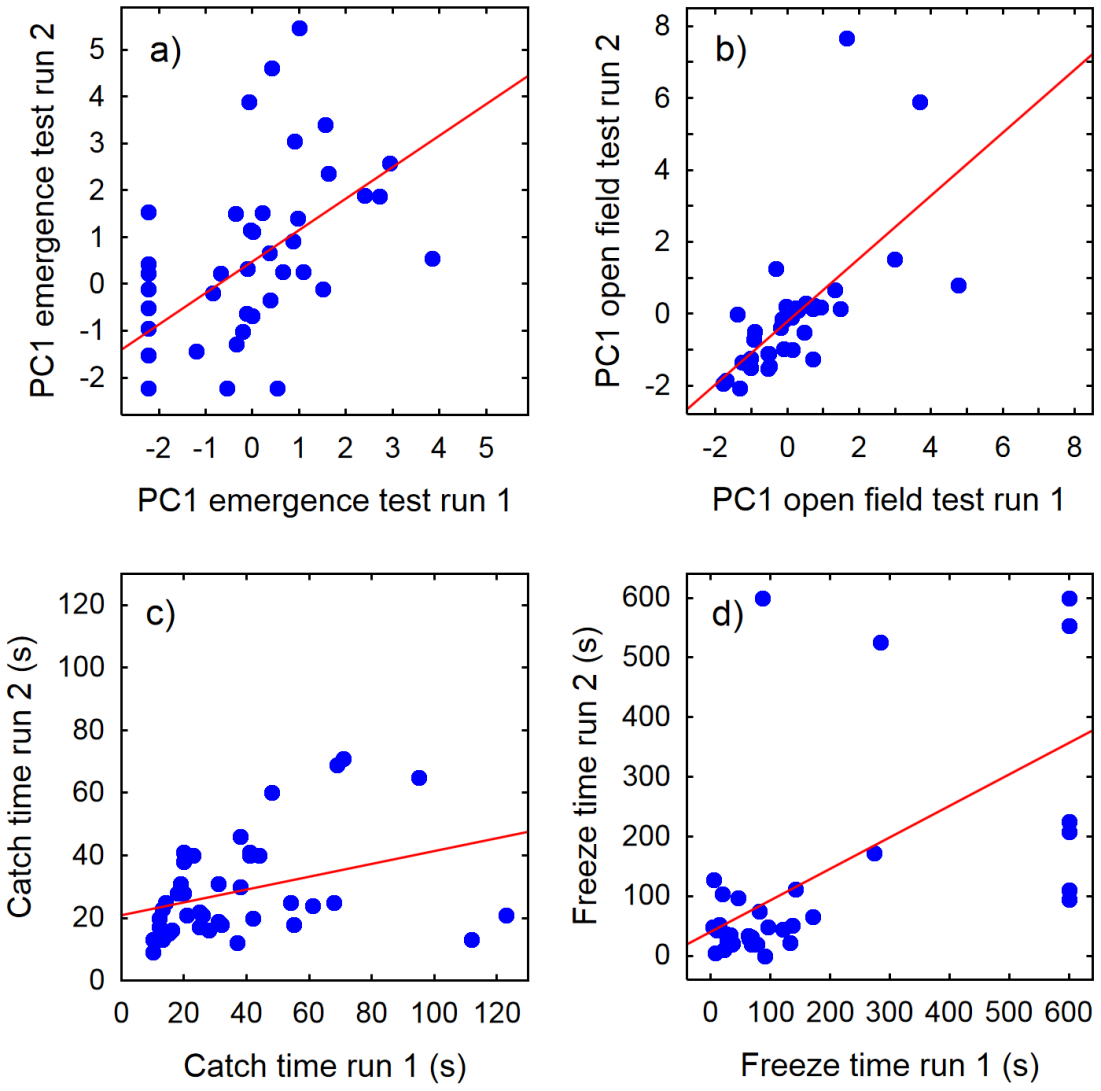
Correlations between behaviour in repeated tests of four boldness assays. a) Emergence test (PC1, r*_s_* = 0.65, p<0.001, N = 44), b) Open field test (PC1, r*_s_* = 0.80, p<0.001, N = 38), c) Catch time (r*_s_* = 0.41, p* = *0.007, N = 42), and d) Freeze time (r*_s_* = 0.55, p<0.001, N = 36) in two-spotted goby males.

In both the emergence test and the open field test, the individual boldness scores (PC1) from the two repeated runs of the same test were strongly correlated (Spearman rank correlation; Figure 2). A strong correlation was also found between the two observations of the latency to start moving after food was distributed in the aquarium (freeze time) (Figure 2). Catch time was measured four times for each individual. The two first catching occasions were significantly correlated (Figure 2), whereas there were no significant correlations between these and later catch times, or between catch times 3 and 4 (p = 0.08–0.63, test details not shown).

### Between-context comparisons

**Figure 3:**
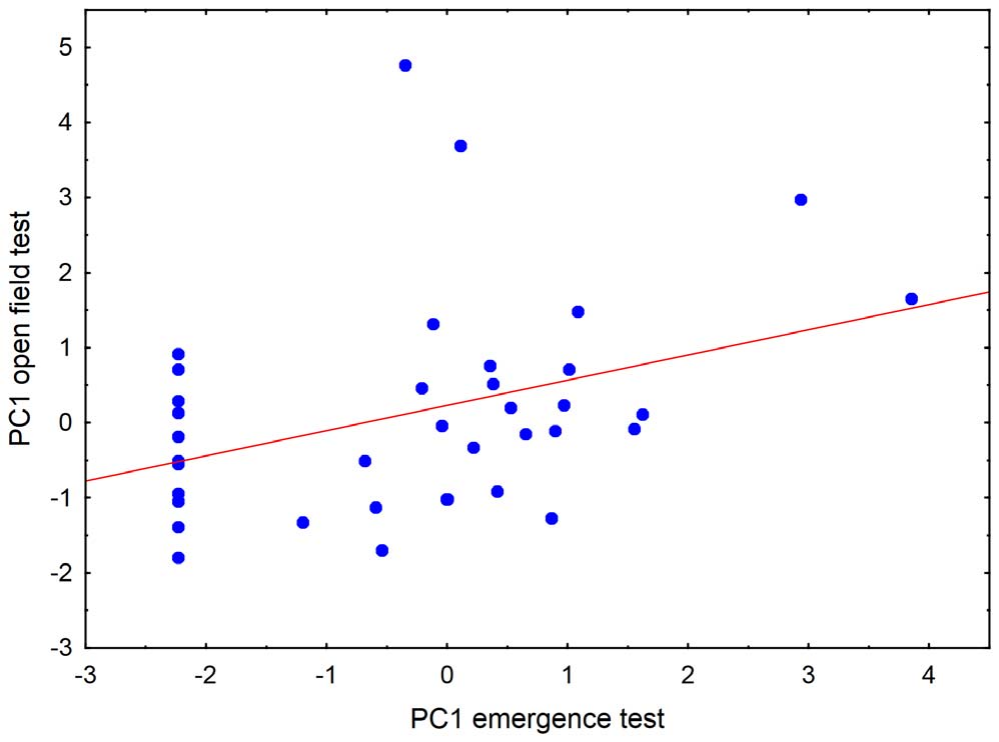
Correlation between individual behaviour in two different assays. Boldness scores (PC1 from the first run), calculated from principal component analyses, of two-spotted goby males tested in an emergence test and an open field test, respectively (r*_s_* = 0.39, p = 0.015, N = 38).

Individual boldness scores from the emergence test and open field test (PC1 for the first run), were positively correlated (Spearman rank correlation; Table 3; Figure 3). Furthermore, catch time was negatively correlated with the boldness score in the assay that directly followed the measurement of catch time, both for the emergence and for the open field test (Table 3), with bolder fish taking shorter time to catch. Also freeze time after food delivery was negatively correlated with PC1 for both the emergence and the open field tests, with bolder fish starting to move sooner after food was dropped into the aquarium compared to shyer fish (Table 3). Freeze time was positively correlated with catch time (Table 3). The correlations between the various assays were all around r_s_ = 0.4, and statistically significant after applying the Benjamini-Hochberg procedure for controlling false discoveries (see Methods for threshold P-values).

**Table 3:** Spearman rank correlations between behaviour estimates from the first runs of four behaviour assays to test boldness in male two-spotted gobies.

	PC1 open field			Catch time			Freeze time		
	r_s_	p	N	r_s_	p	N	r_s_	p	N
PC1 emergence test	0.39	0.015	38	−0.41	0.004	48	−0.44	0.017	48
PC1 open field test				−0.39	0.023	34	−0.35	0.031	38
Catch time							0.39	0.005	48

### Effects of time of season and repeated run

**Figure 4:**
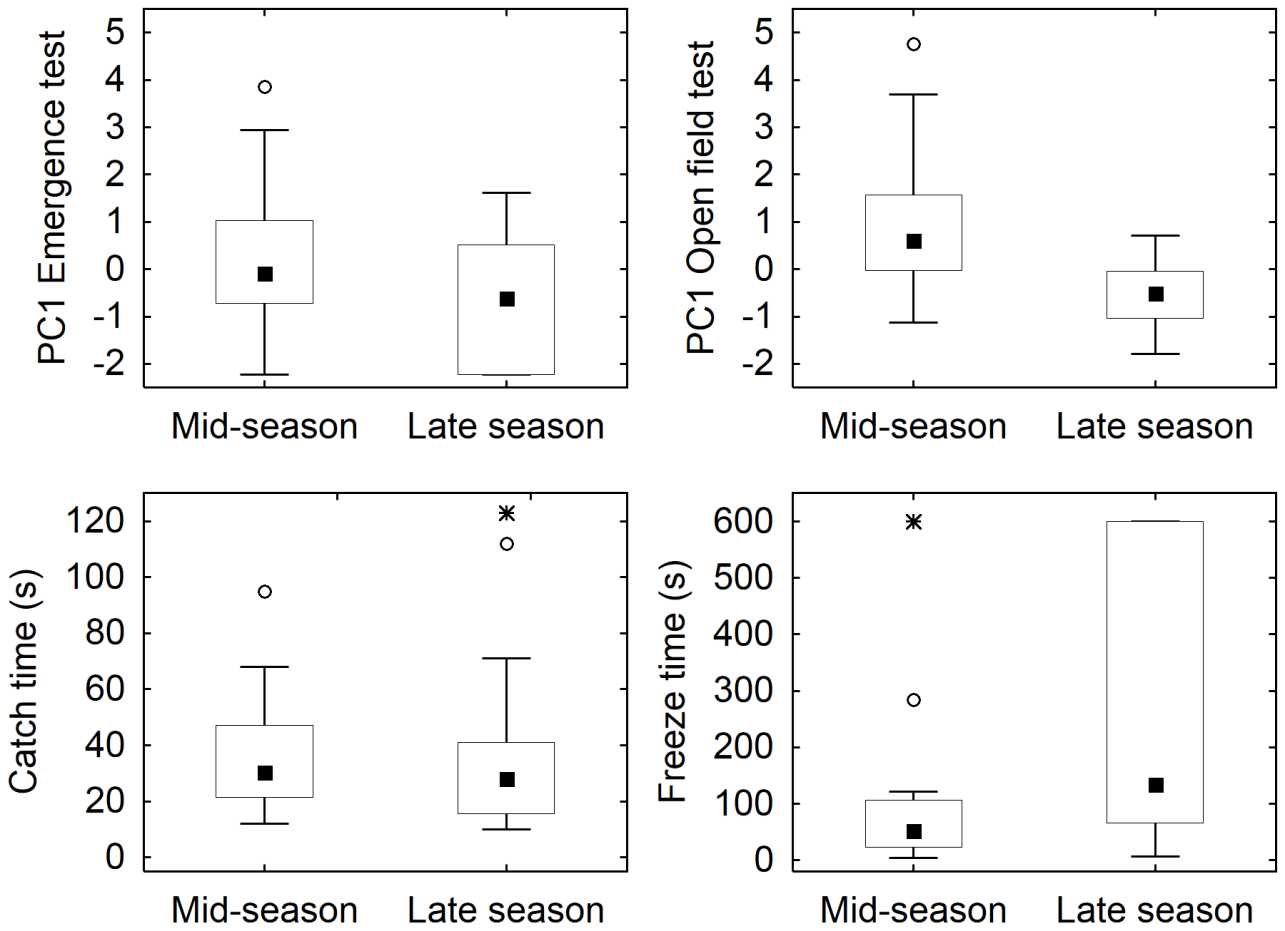
The effect of time of season and run order on boldness in two-spotted goby males. a) Boldness scores (PC1) from the emergence test, b) Boldness scores (PC1) from the open field test, c) Catch time (s), and d) Freeze time (s). Boxplots show median, quartiles, and non-outlier range.

In the open field test, the mid-season set of fish showed a significantly higher boldness than those tested late in the season (Mann-Whitney; Table 4, Figure 4). Freeze time was shorter, that is, indicating higher boldness, in mid-season compared to in late season. We detected no difference in boldness between mid-season and late season for the emergence test or for catch time (Table 4; Figure 4).

**Table 4:** Mann-Whitney tests for the behaviour differences observed during mid and late season, respectively, for the first run of four behaviour assays.

	N/time of season		*Z*	p
	mid-	late		
*run 1*				
PC1 emergence test	24	24	1.39	0.16
PC1 open field test	16	22	3.21	<0.001
Catch time	24	24	0.71	0.48
Freeze time	24	24	−2.29	0.021

When comparing individual scores for the repeated runs, there were significant differences in all assays, except for freeze time (Wilcoxon matched pairs test; Table 5). For the emergence test, the score increased from the first to the second run, whereas for the open field test the scores decreased. Catch time decreased with catching order, from the first to the fourth catching occasion (Friedman's Anova; Table 5). Male size differed between seasons, being slightly larger in the late season. However, of the behaviour assays performed, only freeze time was significantly (and positively) correlated with body length (Spearman Rank, r_s_ = 0.34, p = 0.019), probably reflecting the longer freezing time in late season. PC1 from the emergence test (R_s_ = −0.19; p = 0.21) and the open field test (r_s_ = −0.25, p = 0.13), and catch time (r_s_ = −0.04; p = 0.78) were not correlated with body length.

**Table 5:** Wilcoxon pair-wise comparisons* testing for behaviour differences between repeated runs of four behaviour assays, data pooled for mid-season and late season.

run 1 *vs* run 2	N	*Z*	p	Differences
PC1 emergence test	44	1.96	0.049	1<2
PC1 open field test	38	2.73	0.006	1>2
Freeze time (s)	43	1.61	0.106	-
Catch time (s)	28	9.00*	0.029	1>3,4; 2,3>4

*Catch time was recorded four times and for these comparisons we used Friedman nonparametric tests of multiple dependent variables (χ^2^), using only those fish that were measured all four times. Pairwise comparisons for all four measures of catch time were subsequently tested with Wilcoxon.

## Discussion

Our study showed strong correlations both within and between four different measures of boldness in male two-spotted gobies. The two standard assays used, the emergence test and the open field test, distributed individual fish along the bold-shy gradient in a similar way. These tests were also correlated with the time it takes to catch a fish and how long a fish stays immobile after disturbance. Thus, the relatively consistent ranking of individual boldness across four different assays suggests that boldness is a deeply rooted personality dimension in two-spotted gobies, rather than a context-dependent behaviour. In these small fish, variation in boldness may affect a suite of behaviours, including feeding, mating and responses to predators. Burns (2008) [13] tested the validity of three tests of personality (here termed temperament) in the guppy (*Poecilia reticulata*). He found that both an emergence test and an open field test displayed at least moderate levels of consistency between runs. Furthermore, there were correlations between behaviours measured in these two types of assays. The validity of a third assay, the novel-object test, was lower in that study. Burns (2008) [13] recommended the open field test for the study of boldness, considering that this assay was the one that best measured this personality trait. In a comparison of four different tests of fearfulness in Japanese quail (*Coturnix coturnix japonica*) including an emergence test, Miller et al. (2006) [8] found high consistency within assays, but low between assays. In the three-spined stickleback (*Gasterosteus aculeatus*), latency to feed after disturbance was correlated with shoaling tendencies and shoal position, behaviours that are considered related with boldness in social fish [18]. Also, similar to our study, the order in which the sticklebacks were net-caught from their holding aquarium prior to experiments was negatively correlated with the time spent out of cover during tests, and the order in which fish were caught in two capture events was positively correlated [44].

The comparison of studies on boldness across species is complicated, both by different definitions of the term boldness and by the use of different sets of boldness assays in each species [17]. The use of the same set of assays would facilitate comparisons of boldness across species, and also general inferences about boldness in animals from such studies. At the same time, the assays must obviously be behaviourally and ecologically relevant for the species tested. For a male goby, to leave a shelter for a more exposed area in the emergence test would be comparable to the natural behaviour when leaving his nest site, or other sheltered position among the seaweed, for courtship, fending off rivals, or feeding. The individual balance of gains and risks would reflect personality. We believe that this test is the one with the clearest ecological relevance for the study species, as it directly reflects a choice individuals are frequently faced with in the wild [39]. Similarly, quail entering a novel arena from a familiar pen would trade between safety and feeding opportunities [8].

Even though individual behaviour in our study was correlated between repeated runs for a certain assay, this does not imply that individuals are completely fixed in their behaviours. Significant within-individual differences over repeated runs were found. Thus, instead of a high intra-individual repeatability of measures, we could here talk about rank-order consistency, in which the boldest individuals are always the boldest and vice versa, even though the actual scores change from time to time, as discussed by Schuett et al. (2010) [25]. In the emergence test, individual boldness scores tended to increase from the first to the second run. Also, catch time decreased over repeated observations, indicating habituation to the test situation. In contrast, boldness scores decreased from the first to the second run in the open field test. The changes in behaviour between runs may partly been explained by habituation, but the differences in direction is difficult to explain. Habituation has commonly been reported in other studies of animal boldness [47]–[49]. Thus, experience must be considered when classifying individuals along behaviour gradients. Repeatability of behaviour may differ among taxa and in different environments, as revealed in a meta-analysis of a large number of studies [50], but the effect of habituation will also affect these estimates. Furthermore, combined studies of animal personality and individual plasticity can show that animals of different behaviour type also differ in their behavioural reaction norms [51].

The gobies in this study varied considerably in their degree of boldness. During the breeding season male mating success depends on the ability to attract females to his nests to spawn. Boldness, as measured by our assays, could be highly relevant for a male's likelihood to get mated, as suggested in [25], [52]. The emergence test would mimic a situation when a male leaves its nest, often sheltered within the kelp forest, to search for and court females. In the open field test, with only the aquarium walls as ‘protection’, the male probably would be most visible to potential mates in the central part of the arena.

Boldness may be a sexually selected trait as proposed by Schuett et al. (2010) [25], if mates are chosen according to personality. This is likely to occur in some cases, partly as a consequence of boldness affecting male courtship of females or contests with rival males. In guppies, females prefer to mate with bold males [23]. Bold collared flycatcher males, *Ficedula albicollis*, bonded with a female faster than did less bold males [24]. Furthermore, female zebra finches, *Taeniopygia guttata*, preferred exploratory males over non-exploratory, but only if they themselves were at least moderately exploratory [53]. An intense courtship activity may indicate boldness, but also lead to a lower vigilance and a higher visibility that increases vulnerability to predation. Thus, there can be a trade-off between the chance of finding a mate and avoiding being detected by a predator [22], a likely scenario for the two-spotted goby males. Depending on social and environmental conditions, such as densities of females and predators, respectively, the most successful phenotype may differ across areas and over time, and a variation of personality types could thus be retained in the population. If intensity of sexual selection is varying with time, as found in the two-spotted gobies, this could be one mechanism that maintain variation in boldness.

Males were bolder in the middle of the breeding season than in the end of the season, as revealed by the open field test and the freeze time. Such a seasonal difference in boldness is in line with the temporal change in sex roles found in this species [31], [32]. Male-male competition for females is most intense in the beginning of the season, when there are more males than females ready to mate, and competition then decreases over the breeding season [31]. In the later parts of the breeding season, females, ready to mate, are far more abundant than males, and females compete by courtship and other means for access to males. As a consequence, bold behaviour in the males may be important in mate competition in the early breeding season, as, for example, expressed in active courtship in the open environment [32]. At the end of the breeding season, the few remaining males are typically surrounded and courted by multiple females, and do not need to be bold in order to reproduce [31], [32].

This scenario may suggest that individual males show some extent of plasticity, resulting in decreasingly bold behaviour over the season. However, as we did not follow individual males over the season, our study was not designed to test for this. The behaviour observations for each set of males were carried out over nine days and we can thus only document a behavioural consistency for that time period.

Another, not mutually exclusive explanation, is that the higher boldness seen in mid-season compared to late season is related to differences in life-history trade-offs. The trade-off between current and future reproduction may result in populations with a variation in risk-taking behaviour [26]. Life-history trade-offs were suggested to explain why males of the grey mouse lemur (*Microcebus murinus*) showed increased boldness with age, reflecting the change in the ratio between current and future expected reproductive success [14]. Similarly, nest guarding common goby (*Pomatoschistus microps*) males showed higher risk-taking behaviour in the end compared to earlier in the breeding season [27]. The shift in risk-taking behaviour with time is the opposite in the two-spotted goby, but in this species the changes in male-male competition and survival rates may explain this difference. Bold males may be more successful in mating competition early in the season, but may face a lower survival rate due to costs of reproductive behaviour or boldness in itself [52]. We do find a marked decline in male density over the season in the wild [31]. Thus, the reduced boldness in late season might be explained by a higher survival rate of shy males that have earlier been less reproductively successful. In young trout (*Salmo trutta*), survival in the wild was related to individual personality, and an interaction between natural selection and behavioural plasticity was suggested to affect behaviour [54]. At present, we cannot distinguish between individual plasticity and different life history strategies as potential explanations for the change in boldness.

In conclusion, we have shown that boldness in two-spotted goby males, as quantified by four behavioural assays, seems to be a deeply rooted part of individual personality. With all four assays being correlated, and with correlations for repeated tests of the same assays, there is strong evidence for consistent individual differences along the bold – shy gradient in this model species. Finally, we found an effect of season, with males being bolder earlier in the season compared to later. This may suggest that boldness matches the level of sexual competition at various stages of the season and hence, that personality can be affected by central processes of sexual selection.
